# Practical actions for fostering cross-disciplinary global health research: lessons from a narrative literature review

**DOI:** 10.1136/bmjgh-2020-002293

**Published:** 2020-04-30

**Authors:** Yan Ding, Justin Pulford, Imelda Bates

**Affiliations:** Centre for Capacity Research, Liverpool School of Tropical Medicine, Liverpool, UK

**Keywords:** review, public health, health systems

## Abstract

**Introduction:**

Global health research involves disciplines within and beyond the health sciences. A cross-disciplinary collaborative research approach enables an interchange of knowledge and experience and stimulates innovative responses to complex health challenges. However, there is little robust evidence to guide the design and implementation of cross-disciplinary research in global health, hampering effective collective action. This review synthesised evidence on practical actions for fostering cross-disciplinary research to provide guidance on the design and implementation of research in global health.

**Methods:**

We searched five electronic databases using key words. The search included original research and research notes articles in English. We used a framework adapted from the socio-ecological model and thematic synthesis for data analysis.

**Results:**

Thirty-six original research and 27 research notes articles were included in the review. These were predominantly from high-income countries and indicated that practical actions on fostering cross-disciplinary research are closely linked to leadership and teamwork which should be planned and implemented at research team and institutional levels. The publications also indicated that individual qualities such as being receptive to new ideas and funders’ power and influence have practical implications for conducting cross-disciplinary research. Practical actions that individuals, research team leaders, academic institutions and funders can undertake to foster cross-disciplinary research were identified.

**Conclusion:**

Our review found evidence from high-income countries, not low-and-middle-income countries, about practices that can improve cross-disciplinary research in global health. Critical knowledge gaps exist around how leadership and teamwork processes can better integrate expertise from different disciplines to make cross-disciplinary research more effective.

**Key questions**

**What is already known?**

- Global health can be advanced by cross-disciplinary collaboration within and beyond the health sciences.
- Designing, implementing and evaluating cross-disciplinary research, including for global health, faces challenges, which hampers effective collective action.
- Information on the enablers and barriers of cross-disciplinary research are fragmented across academic disciplines.

**What are the new findings?**

- Existing published evidence on fostering cross-disciplinary research in practice is mainly from high-income countries.
- Practical actions for fostering cross-disciplinary global health research are closely linked to leadership, management, collaboration and teamwork.
- Individual qualities such as being receptive to new ideas, dealing with the unknown, commitment and confidence, as well as funders’ power and influence, all have practical implications for conducting cross-disciplinary research.

**What do the new findings imply?**

- Critical knowledge gaps on fostering cross-disciplinary research on global health exist for low-and-middle-income countries.
- Individual researchers, research team leaders, academic institutions and research funders, building on the practical examples from this review, can improve cross-disciplinary research in global health.

## Introduction

Global health is “a collection of problems” which “turn on the quest for equity”.^1 2^ Global health encompasses prevention and treatment, and emphasises transnational health issues, determinants and solutions.^3 4^ Solutions to complex global health research problems depend on effective research collaborations between disciplines within and beyond health sciences and multiple sectors of society.^3 5 6^ A cross-disciplinary approach enables an interchange of knowledge and experience,^7–12^ stimulates innovative responses to complex societal challenges,^13–20^ and plays an important role in translating and disseminating knowledge into practice and policy.^21–23^

The term ‘cross-disciplinary research’ is used to cover three typologies—multi-disciplinary, inter-disciplinary and trans-disciplinary research—which are in the continuum of collaboration.^24–31^ This paper defines cross-disciplinary research as one that combines and, in some cases, integrates concepts, methods and theories drawn from two or more disciplines. Our focus is on cross-disciplinary research in teams, though we acknowledge that an individual could also conduct cross-disciplinary research.

Designing, implementing and evaluating cross-disciplinary research, including for global health, faces challenges.^3 6^ Cross-disciplinary research does not have its own epistemology,^3 19^ and has more uncertainties in research processes and outcomes than single-disciplinary research.^21 32 33^ Although cross-disciplinary integration is a central theme, there is no agreement on its meaning^30 34 35^ and there is limited published guidance to enable integration.^30 36 37^ The peer review process for grant proposals and journal articles of cross-disciplinary research is challenging^38–40^ with no existing quality standards nor guidelines for evaluation.^39 41^

The challenges for cross-disciplinary research in global health include problem definition and positioning. This results in problems and solutions being conceptualised in varied ways in different disciplines, hampering effective collective action,^42^ and in poor co-ordination of effort in cross-disciplinary research.^43^ Greater use of management science in global health, synergistic interactions between individuals, community and national actors have been proposed to address these problems.^44^

Information on the enablers and barriers of cross-disciplinary research are fragmented across academic disciplines.^20 45^ We are only aware of five reviews on this topic. Three did not review empirical studies.^33 46 47^ One reviewed cross-disciplinary research implementation^48^ especially the growth of trans-disciplinary sustainability research, the methods adopted and the engagement of practitioners, but did not cover barriers and enablers of cross-disciplinary research.^48^ One summarised four groups of factors that influence the trans-disciplinary research process, including personal attitude, communication culture, skills and knowledge, and project structure,^20^ but such factors were not a main component of the review.^20^ It is the lack of strategic communication and collaboration plans across the studies that were reviewed.^20^ A review of empirical studies focusing on the barriers and enablers of conducting cross-disciplinary research is needed to address this knowledge gap. The purpose of this narrative review was therefore to synthesise the evidence on practical actions for fostering cross-disciplinary research in order to provide guidance on the design and implementation of cross-disciplinary global health research.

## Methods

### Search strategy and inclusion criteria

We searched five electronic databases (MEDLINE, CINAHL COMPLETE, Global Health, PubMed, Web of Science) using key words combined with the Boolean operators (AND, OR) up to 31 December 2018, and with no start date (box 1), limited to original research or research notes articles in English (box 2). Abstracts from all potentially eligible studies were reviewed, followed by full-text screening if indicated. We scanned the reference lists of the included studies for relevant articles. As global health involves disciplines within and beyond health sciences, our search strategy and inclusion criteria cover all disciplines.

Box 1 **The terms for the literature search.**

[(multi-/inter-/trans-/cross-disciplinary research/study)

OR

(inter-professional/multi-institutional collaboration/partnership)]

AND

[(pitfalls/obstacles/difficulties/barriers/challenges/constrains/drawbacks/ disadvantages/enablers/facilitators/opportunities/advantages)

OR

(definition/process/strategies/theory/framework(s)/model(s)

OR

(evaluation/assessment/appraisal/efficiency/effectiveness/quality/sustainability)]

Box 2 **Inclusion criteria for publications.**

**Original research or research notes articles**

Those that describe/analyse enablers, barriers, strategies or activities of cross-disciplinary research with cross-disciplinary research as a main research topic.

**Definition of original research:**

Publications in which (1) a hypothesis, research question or study purpose was stated, (2) research methods described, (3) results reported, and (4) the results and their possible implications discussed.^131^

**Definition of research notes articles:**

Scientifically valid research outputs that cannot be considered as full research,^132^ [since they do not provide a] deep understanding of the actors, interactions, sentiments, and behaviours occurring for a specific process through time as the principal objective.^133^

### Data extraction and analysis

**Figure 1:**
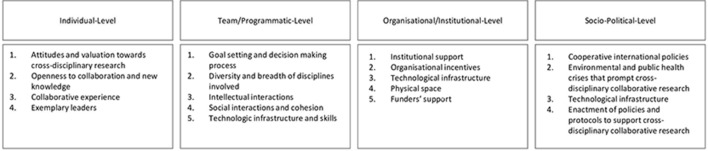
Analytical framework for this narrative literature review.

All eligible original research and research notes articles were read in full. To identify and analyse practical actions in conducting cross-disciplinary research, we constructed an analytical framework (figure 1), extracted the relevant information and mapped it to the appropriate section. Using thematic synthesis, we produced a narrative summary of the information. We adopted thematic synthesis to address questions on the need, appropriateness, acceptability and effectiveness of an intervention through an inductive approach using a ‘constant comparison’ method.^49^ The analysis focused on identifying and distilling practical actions that foster cross-disciplinary research for global health. We provided a summary of the key actions along with the number of publications that mentioned each action, to give a sense of the weight of evidence for each action.

The socio-ecological model on which this framework is based incorporates six contextual factors; intrapersonal, interpersonal, organisational/institutional, physical/environmental, technological and socio-political.^33^ We adapted this model by combining technological and physical/environmental factors under organisational/institutional, as technology and physical environment are organisational resources.^49^ We replaced intrapersonal with individual and changed interpersonal to team/programmatic. The 18 headings displayed in the analytical framework were adapted from the original framework and were revised to make them concise.

### Patient and public involvement

Patients and public were not involved in the design, conduct or reporting of this narrative review.

## Results

### Search results and eligible publications

**Figure 2:**
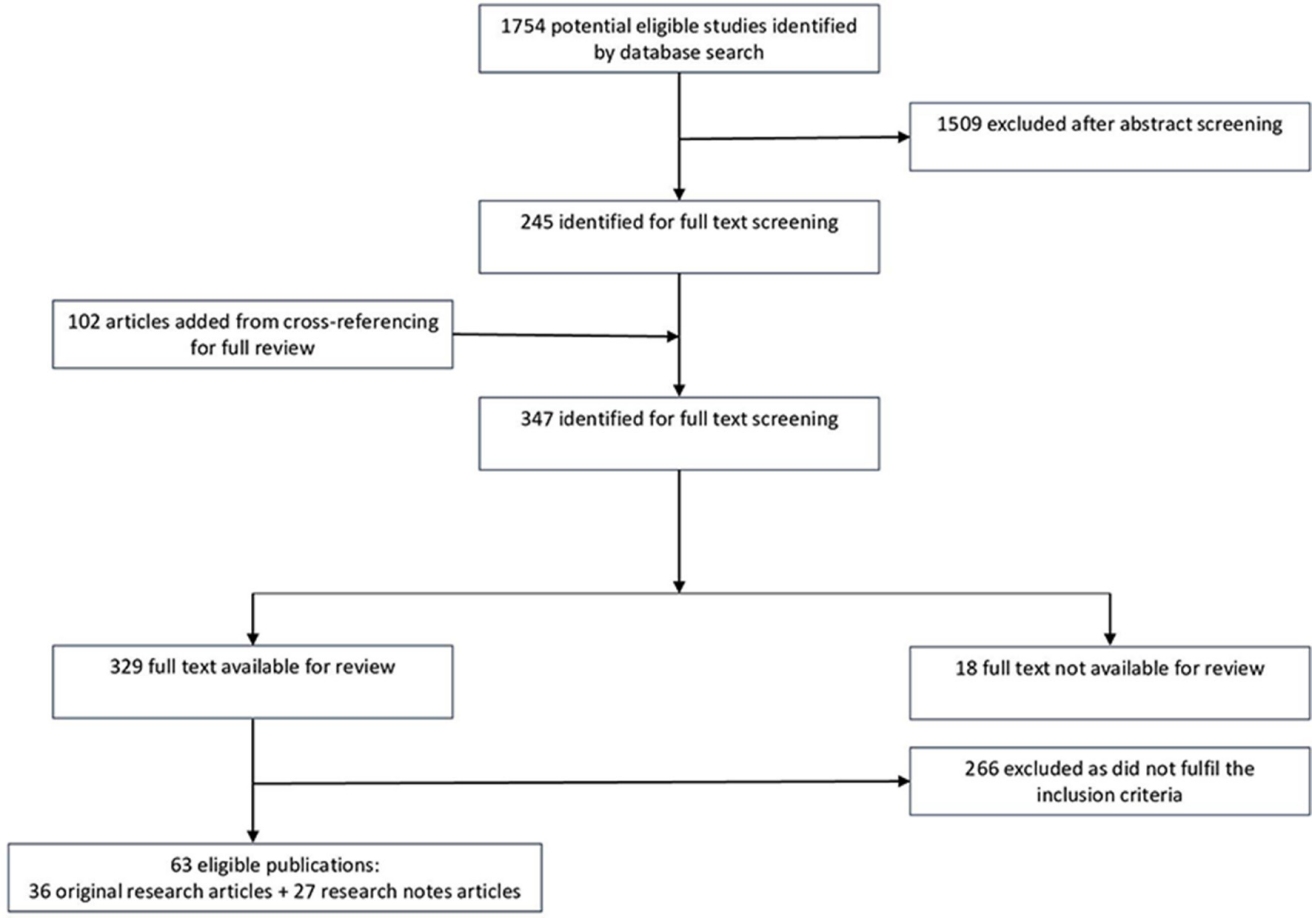
Flow chart for the search and selection process for eligible publications.

A summary of the literature search, selection process and results is provided in figure 2. An overview of the included publications is provided in online supplementary file 1. Publication of the original research (36) and research notes articles (27) predominantly started in 2000 (62 of 63). The studies used qualitative research methods, especially interviews and thematical analysis, more frequently than quantitative ones, though many studies did not explicitly state their data collection methods (22 of 63) and/or data analysis methods (29 of 63). Fifty-one studies occurred in high-income countries (based on World Bank income categories).^50^

10.1136/bmjgh-2020-002293.supp1 Supplementary data

### Practical actions for fostering cross-disciplinary research

Practical actions considered important for participating in cross-disciplinary research were described in the literature at the individual, research team/programmatic and institutional/funder level (table 1). We found no relevant practical actions at the socio-political level.

**Table 1:** Practical actions for fostering cross-disciplinary research and the number of publications that mentioned each action

Practical actions for fostering cross-disciplinary research (CDR)	Number of publications that mention each action (n=63)
**At the individual level**	
**1. Personal attributes**	**25**
1.1 Receptive to new ideas and willing to learn from others	17
1.2 Courageous to push disciplinary boundaries	7
1.3 Dealing with the unknown	4
1.4 Aware of and coping with negative emotions generated while conducting CDR	10
**2. Motivation to conduct CDR**	**10**
2.1 A strong belief in the added value of CDR	8
2.2 Creative outputs and better theories and analyses generated through CDR	5
2.3 Better understanding of one’s own disciplines by doing CDR	1
**3. Commitment and confidence in teamwork**	**15**
3.1 An individuals’ willingness to work collaboratively	8
3.2 Individual commitment to conduct CDR	8
3.3 Individuals’ confidence to explore and initiate CDR	4
**4. Career progression**	**4**
4.1 A large publication record within a primary discipline	3
4.2 The continuity and development of CDR networks and communities	1
**At research team/programmatic level**	
**1. Good leadership**	**26**
1.1 Explicit knowledge integration goals	3
1.2 Integrative and clear vision	12
1.3 Leaders’ personal quality Trustworthiness, transparency and openness Recognising complementary expertise, understanding differences and managing expectations Communication skills, team-building skills	10
1.4 Organising and expanding collaborative networks	5
1.5 Disengaging partners that cannot find ways to work together productively	4
**2. Establishing a cross-disciplinary team**	**23**
2.1 Clearly identified roles	4
2.2 A balanced team of experienced and early-career researchers	2
2.3 A central administration team providing leadership and administrative support	5
2.4 Research brokers to facilitate communication among disciplines	8
2.5 Collaborations based on pre-existing networks	11
**3. Working as a cross-disciplinary team**	**42**
3.1 Defining and framing research problems collaboratively	5
3.2 Working to a common conceptual framework	8
3.3 Conflict prevention and management through communication and open discussions, by internal agreed approaches, and turning competing demands into opportunities for growth	15
3.4 Identifying and minimising academic and discipline hierarchy	11
3.5 Engaging local stakeholders, especially through a continuous participatory approach, joint field trips and with the support of a communications specialist	6
3.6 Mentoring early-career researchers	6
3.7 Nurturing trust within CDR teams and from funding agencies and hosting institutions	12
**4. Cross-disciplinary communications**	**32**
4.1 Constructing a shared understanding with developing a shared language as a milestone, through mutual learning, and by team-level reflection	19
4.2 Having regular meetings, from informal ones to formal ones, either in-person or through virtual meetings and electronic communication	27
**At institutional/funder level**	
**1. Institutional support**	**10**
1.1 Promoting a CDR culture	2
1.2 Establishing institutional structures such as CDR centres	2
1.3 Creating a common administration infrastructure	5
1.4 Initiating and maintaining CDR mentorship schemes	4
1.5 Disseminating CDR funding information	2
1.6 Facilitating networking and matching research collaborators	4
**2. Academic career pathways**	**6**
2.1 Structuring and implementing faculty incentives valuing CDR appropriately	6
**3. Providing institutional resources**	**21**
3.1 Institutional seed money	10
3.2 Meeting venues and tools for research management	5
3.3 Shared space, that is, offices, buildings, campuses, study sites	14
**4. Funders’ power and influence**	**16**
4.1 Dedicated funding for CDR, especially long-term and seed funding, or by promoting CDR in funding calls	9
4.2 Commission research on CDR communication and co-ordination	4
4.3 Flexible review processes for funding applications	4
4.4 Linking researchers across disciplines	4
4.5 Engaging with universities and publishers for better recognition of CDR	1
4.6 Engaging policy makers when the research is policy relevant	1

### Practical actions for fostering cross-disciplinary research at individual level

#### Personal attributes

##### Receptive to new ideas

Individual qualities such as open-mindedness to other disciplines and learning are valued in the selection of cross-disciplinary team members.^51–53^ Individuals’ awareness of the various disciplinary assumptions, concepts and methods underline cross-disciplinary collaborations.^54^ Such awareness can be obtained by attending events and through exposure to different communities.^55^ Researchers’ willingness to explore and learn new ideas, knowledge and perspectives,^30 31 36 38 40 52 54 56–62^ and to share theirs,^60 62^ coupled with accepting their disciplines’ limitations^57 60^ are enabling factors. The courage in negotiating and pushing one’s own disciplinary boundary by being explicit and flexible is valued when working in ways that are not necessarily legitimated by one’s own disciplines.^8 30 38 57 60 61 63^

##### Dealing with the unknown

The ability to rapidly digest information and its implications is helpful for cross-disciplinary working.^8 40^ Researchers should be mentally prepared for unexpected results,^61^ and to allow for critical self-reflection on assumptions of involved disciplines and the decision-making processes.^64^

Negative emotions are common in cross-disciplinary research^26 38 56 57 65 66^ and often last throughout the whole research process.^57 65^ Emotional difficulties occur due to unfamiliarity with such research process^56 67^ which force researchers to enter into unfamiliar areas experiencing feelings of anxiety, insecurity and incompetency.^38 57 58 60 66^ When new theories conflict with researchers “internalised rational academic norms and intellectual values”, researchers can experience “frustration of in-coherence”.^68^ A trusting and supportive cross-disciplinary research community helps overcome emotional difficulties.^38 65^

#### An individuals’ commitment and confidence in teamwork

A teamwork approach is essential for cross-disciplinary working including a willingness to work collaboratively^30 38 40 59 60 69 70^ with a strong belief in the added value of such research.^31 36 38 54 55 63 66 68^ Maintaining individual commitment to conduct cross-disciplinary research throughout the research process is frequently emphasised in the literature, and commitment at the beginning is important^29 31 38 56 60 66^; for example, devoting time to cross-disciplinary networking^29 52 60^ and learning about others’ research perspectives and approaches.^52 54 60 66^ Appreciating others’ efforts in working together is key for individual inclusion in a cross-disciplinary team.^62^

Individuals need to understand the continuum of collaboration and the typologies of cross-disciplinary research.^56^ Their confidence to explore and initiate cross-disciplinary research is important for success.^31 54 55^ Those experienced in conducting inter-disciplinary and trans-disciplinary research are more capable and confident in describing the various epistemologies they encounter.^54^ The degree of individual self-confidence in conducting cross-disciplinary research is loosely correlated with their career stages and the accumulation of experience on cross-disciplinary collaborative research.^52^ Longevity of work experience, and experience in other universities, firms and strategic disciplines (ie, basic research which produces a broad base of knowledge to solve practical problems) are positively related to willingness to undertake cross-disciplinary research.^71^

#### Assessing the benefits to the individual

Individuals’ research careers tend to progress better if they engage in single-disciplinary research instead of cross-disciplinary collaborative research,^22 30 36 38 40 59 71^ and this may influence their willingness and commitment to continue cross-disciplinary working.^40^ One of the best motivators for developing and maintaining a cross-disciplinary research is the creative outputs and better theories and analyses generated through cross-disciplinary research.^8 31 38 66 72^ The benefits of such research extend to researchers’ better understanding of their own disciplines.^54^ Early-career researchers can gain cross-disciplinary research experience through networking events^73 74^ and by identifying institutions and mentors favourable to cross-disciplinary research.^74^ Researchers’ future career options can be enhanced by the continuity and development of cross-disciplinary research networks and communities.^75^ A large publication record within a primary discipline is good for securing a permanent job while still being able to undertake cross-disciplinary research.^29 53 74^

### Practical actions for fostering cross-disciplinary research at team/programmatic level

#### Good leadership

##### Personal qualities

Good leadership is critical,^9 22 26 29 56 66 69 70 75–78^ as the quality of cross-disciplinary research lies in how disciplines are brought together.^74^ Good leaders should have explicit knowledge integration goals^37 59 67^ and recognise complementary expertise^54 68^ through a sufficient understanding of research topics and disciplines.^36 54^ They should identify differences across disciplines and researchers, facilitate discussions on the implications of these differences,^78^ and manage expectations by identifying the limitations of disciplines.^52^

The diverse nature of cross-disciplinary research teams increases the chance of dual lines of leadership—one by discipline and one by the research team.^30^ Management thus requires a professional leader with personal compatibility,^54^ mutual respect and trust.^52 60^ Trust is built by leaders who maintain fairness in the recognition and reward of team members’ contributions.^54^ Therefore, leaders’ personal qualities, including trustworthiness, transparency, and openness to different approaches and perspectives, have been shown to encourage and influence team members.^53 61 70 78^

##### Integrative and clear vision

Integrative vision means keeping sight of the goals and aligning the respective scientific interests of research team members.^22 26 66 75 76 79–81^ A leader’s vision for the cross-disciplinary research project,^73^ and for the future of the cross-disciplinary research field,^53^ is essential when engaging team members and non-academic stakeholders. Good leaders communicate their vision effectively^53 73^ and catalyse the integration of disciplines with team-building skills.^73^ A clear and shared vision on what a successful cross-disciplinary research project looks like helps harmonise team effort^31 40^ while making the goal of knowledge integration clearer.^26^

##### Network development and evolution

Good leaders are strong in organising and expanding collaborative networks.^29 36 66^ They are able to understand the limitations of their own networks,^55^ to move a research agenda forward,^66^ to build a cross-disciplinary research community,^55^ and to create collaboration opportunities for those not yet working together.^36^ Leaders should bring potential research collaborators together early to agree on research problems.^73^ Reading and discussing key articles together is helpful^60^ and they should not be afraid to disengage from partners that cannot find ways to work together productively,^53 55 62^ or who do not meet expectations^29^ managed through planning for respectful exits.^29^

#### Establishing a cross-disciplinary team

##### Defined roles

Clearly identified roles in the research project from the onset ensure that team members understand what is expected of them and how everyone contributes to the team.^9 26 56 82^ A balanced team of experienced and early-career researchers is effective in facilitating cross-disciplinary research processes,^29 83^ being more collaborative than competitive.^29^ Experienced researchers provide guidance and support, and early-career investigators implement research projects and are supported to publish findings.^29 83^

A central administration team providing leadership and administrative support (ie, hosting annual meetings, workshops and co-ordinating communications) to members of a cross-disciplinary programme is recommended.^83 84^ As the time required for planning and managing cross-disciplinary research projects is high,^36 85^ the role and skills of the central administration team should be valued and supported.^74 84^

##### Research brokers

Individuals who act as ‘brokers’ among disciplines are enablers in cross-disciplinary teams.^8 26 36 55 66 81^ Successful research brokers have the capacity to articulate and communicate disciplinary assumptions and perspectives,^66^ to create networking opportunities and dialogue platforms,^36^ and can see commonalities among disciplines,^81^ or commit efforts to ensure that the initial connections develop into functional collaborations.^55^ Social scientists often take such a role due to their training background.^8 26^ However, social science should not be regarded as a ‘service-discipline’ to facilitate team member interactions.^67^

##### Collaborators

Identifying suitable partners and establishing collaborations can be challenging.^40^ Research partner selection in many cases is based on pre-existing networks,^40 52 60 67 86^ supplemented by informal contacts.^40^ Prior established working relationships facilitate cross-disciplinary collaborative research,^56 59 60 69 85 87^ by enabling trust and rapport to be built quickly,^31 59 85^ and through pre-existing knowledge of ways of working and thinking.^31 60^

#### Working as a cross-disciplinary team

##### Defining research problems and working to a common conceptual framework

Defining and framing research problems collaboratively at the onset is good practice^37 60 64^ to develop joint understanding^37^ and for writing research proposals.^66^ Researchers from various disciplines may interpret a problem differently,^36 55 60 80^ especially when working with non-research partners.^88^ Individuals who read research papers from other disciplines and discuss together,^55^ who explore dimensions to test problem boundaries and who tolerate ambiguity are valuable for identifying optimum research scope and finalising research questions.^40^

A common conceptual framework is vital for a cross-disciplinary research project^60 66 67 72 85 89^; it clarifies the scope of research,^67 85^ shows the possible complex interactions among different variables/factors,^89^ displays contributions of each discipline,^60^ provides guidance for collaboration,^66 67 85^ and facilitates both internal and external communications regarding the overall research project.^66 85^ Continuous efforts in finding common ground for cross-disciplinary research collaborations are appreciated.^55 71^

##### Conflict prevention and management

Competition within a cross-disciplinary research project/programme can occur over the split of resources, workloads, credit (ie, authorship) and the relationship with funders.^31^ Harmonisation can be promoted by the instigation of internal agreed approaches for methods of working, data analysis and authorship,^9 29 31 82^ and good communications and open discussions,^30 40 55 62 82^ especially on publishing and research approaches.^8 54^

Differences across disciplines are vast and include philosophical,^30 31 88^ measurement standards,^51^ framing of concepts,^64^ attitudes to theory and practice,^51^ the use and understanding of terminology,^30 31 56^ and expectations of communication and etiquette.^51 56^ Therefore, cross-disciplinary researchers have to build mutual understanding and discuss acceptable ways forward.^36 40 51 73^ They can immerse themselves in the languages, cultures and knowledge of their cross-disciplinary research collaborators^73^ and engage in frequent informal encounters.^9 23 29 38 52 60 63 68 71 81 85^

##### Hierarchy

A leadership style appreciating and encouraging contributions from various disciplines is particularly relevant to cross-disciplinary research.^81^ Identifying and minimising academic and discipline hierarchy^38 57^ by empowering every team member^56^ and developing an understanding of the contribution of other disciplines^40^ gives recognition to all areas of expertise.^22^ Specific approaches to achieving a hierarchical balance include ensuring each member strikes an equilibrium between leading and following,^73^ and contributing to and benefiting from team efforts^73 81^; undertaking pacing actions to allow time to integrate new members and ideas^29^; and asking early-career researchers for their insights and feedback^38^ to ensure the opportunity to contribute.^78^ This approach allows for accommodating perspectives to arrive at an agreed study design^31 57^ and provides opportunities for interactions.^77^

##### Engaging local stakeholders

Good relationships with stakeholders, especially practitioners and policy-makers, are essential.^86^ They promote ownership and uptake of the research results.^79 83^ A continuous participatory approach instead of occasional consultation meetings enables local stakeholders to gradually take ownership of findings.^90^ Conducting open discussions about motivations and goals avoids unrealistic expectations and subsequent frustrations for both researchers and stakeholders.^86^ Joint field trips between researchers and stakeholders and communication on the ground enables learning about local realities and about the knowledge demands of local stakeholders.^80^ Working with a communications specialist helps such engagement.^84^

##### Mentoring early-career researchers

Experienced researchers hiring early-career researchers from other disciplines should understand their juniors’ expertise and educate them in new specialties.^73^ Cross-disciplinary research leaders should act as role models and mentors to early-career researchers.^53^ Co-supervision is a common approach to train PhD students expanding in multiple disciplines.^52^ Mentorship should be offered to other experienced researchers,^74 91^ as co-supervision from various disciplines can bring faculty members together for cross-disciplinary research.^36 53^

##### Trust

Trust within cross-disciplinary research teams^31 38 56 60 75 92^ and from funding agencies and hosting institutions is important^61^ and can be developed through transparency and stability of research systems and processes^81^; clear and open communication on assumptions, research design, implementation and results^80 85^; and fair recognition and distribution of credit.^54^ Setting realistic shared goals and boundaries is paramount to avoid mistrust.^86^ Shared goals often take the form of ideas for manuscripts and grant applications,^36 37 91^ and mutual learning and working together towards implementable solutions for societal problems.^91 93^

#### Cross-disciplinary communications

##### Shared understandings

Barriers between cross-disciplinary researchers can be avoided by having dialogue to construct a shared understanding and break down disciplinary jargon,^38^ using context when proposing theoretical approaches,^64^ clarifying and posing ‘stupid’ questions,^52 55^ and expressing meanings rather than results to make complex content accessible.^94^ The development of a common language (ie, a collective set of vocabularies that clarify the terminologies of involved disciplines) is a milestone.^38 60 62–64 66 88 95^ Knowledge sharing,^60 62^ listening,^62^ discussions^60 62^ and clarification^38 62 88^ are key enabling processes.

Mutual learning allows researchers to develop respect for colleagues’ expertise in various disciplines.^51^ Facilitating this includes assessing team members’ background to become familiar with the strengths of the team^82^ and motivating team members to teach one another about their respective disciplines^36^; however, to become acquainted with and develop respect for each other’s disciplinary culture may take years.^37^

Team-level reflection on the process and outcomes of cross-disciplinary research allows researchers to examine the underlying assumptions of their perspectives, to intentionally integrate with other perspectives^38 56 57 85^ and to understand the dynamics of cross-disciplinary research projects.^61^ Such reflection may not be practised enough.^91^

##### Meetings

Regular meetings are important to foster cross-disciplinary working^9 23 29 31 38 55–57 59 67 71 80 89 96^ as they promote leaders by affirming their roles,^96^ and allow for communications on the direction and changing context of the studies.^96^ Types of meetings range from informal chats (eg, over meal times and coffee breaks),^9 36 54 55 63^ off-campus retreats, speed dating/networking events,^55^ to seminars/workshops,^29 55 59 71 80 85^ brainstorms^61 94^ and ‘sandpits’ (meetings spread over several days).^61^ Informal meetings increase participants’ comfort levels,^54 68 89^ especially among would-be collaborators,^54^ and encourage creativity.^60^ Meetings are especially useful in times of conflict,^38 56^ ensuring accountability and strengthening working relationships.^38 80 97^ Off-campus retreats are useful to promote open dialogue and trust, to address cross-disciplinary tensions and to facilitate intellectual integration.^9 23 29 38 60 68 71^ Virtual meetings, electronic communication and telephone calls are important and used often in cross-disciplinary research communication,^29 38 56 59 66 98^ although some authors stated that “Technology did not overcome distance”.^98^

#### Fostering cross-disciplinary research at institutional/funder level

##### Institutional support

Academic institutions/organisations^22 56 59 71 73 76 77^ play an important role in fostering cross-disciplinary research. Measures include explicitly incorporating cross-disciplinary research in high-level mission statements,^59^ establishing institutional structures such as cross-disciplinary research centres and thematic networks across departments/faculties,^74 99^ and using senior leaders as cross-disciplinary research champions.^53^ Creating a common administration infrastructure facilitates institutional-wide departments/faculties collaboration^22 54 73 74^ (ie, budgetary and cost-sharing policies^54 73 74^). Meetings between principal investigators and university-level research support staff are useful to overcome administrative difficulties.^59^ These measures are significant midterm institutional changes driven by cross-disciplinary research and the teams.^15 67^

Initiating and maintaining cross-disciplinary research mentorship schemes^74^ and providing cross-disciplinary training through master’s courses and PhD research projects are the groundwork for future cross-disciplinary research,^53 99^ and accelerated through providing institutional funding for cross-disciplinary training.^36^

Dissemination of cross-disciplinary research funding information by university/institute research offices is an enabler,^53^ as is forming task forces on promoting cross-disciplinary collaborations.^59^ Such offices are ideally positioned to match research collaborators^54^ and to facilitate network development through organising institutional-wide meetings/workshops.^53 54 75 76^

##### Academic career pathways

Traditionally, academic tenure and promotion schemes reward individual research instead of teamwork.^36 100^ Cross-disciplinary research team members are mainly evaluated individually.^40^ As cross-disciplinary research takes more time with less recognised value at the individual level,^22 38 56 61 66 71 89 98^ consequently early-career and mid-career researchers have concerns about their engagement in such research.^29 36 38 53 56 59 61 71 74 89 95 99 100^ In addition, cross-disciplinary research teams tend to be more discipline focused in publishing since journals tend to have a single-discipline focus,^101^ which fractures the research synergies constructed in cross-disciplinary research.^38 54^

Structuring and implementing faculty incentives valuing cross-disciplinary research appropriately at the institutional level,^53 54 74 99^ and praising institutional leaders with a strong cross-disciplinary research drive, enable policies to thrive.^59^ Such policies nurture the continuity of cross-disciplinary research networks and communities.^75^

##### Provision of institutional resources

A number of articles highlighted the value of institutional seed money to initiate cross-disciplinary research projects.^22 36 53 59 61 73–75 77 99^ This does allow for funding flexibility^61^ and also enables early-career researchers to become co-principal investigators.^36^ Seed funding is important as cross-disciplinary research takes time and groundwork,^61 74^ and research teams with collaborative experience are more likely to secure funding and deliver outputs in the longer term.^99^

Institutions can provide resources such as meeting venues and tools for research management, as cross-disciplinary research requires long-term data management capacity and resources such as data storage, access and ownership.^36 56 77 85 88^

Physical space design within institutions can foster cross-disciplinary research by creating opportunities for interaction,^51 52 63 71 95^ and for generating mutual understanding and trust.^9 36 52 56 60 68 85 95^ Measures include sharing offices,^51 52 56 59 71 77 99^ sitting in the same building,^52^ working at the same campus^63^ and sharing study sites.^52^

##### Funders’ power and influence

Providing dedicated funding for cross-disciplinary research is the ultimate enabler that funders provide.^38 60 61 101 102^ A flexible, hands-off management style allows for creative solutions.^61 68 75^ Long-term funding (ie, ≥3 years^95 101^) can support larger research questions and provide time to define shared cross-disciplinary research problems. Adopting multi-stage funding models for the development of research consortia avoids wasting resources.^40 83 101^ Offering seed funding and allowing early-career researchers to be co-principal investigators nurtures cross-disciplinary research communities.^36 102^

In addition, funders are in a position of power to promote cross-disciplinary integration in funding calls,^36 40 63 101 102^ to commission research on cross-disciplinary communication and co-ordination,^9 36 40 98^ and to allow for flexible review processes recognising that cross-disciplinary research projects need their own project-specific metrics or review process.^72 95 103 104^ The intellectual breadth of cross-disciplinary research proposals is appreciated by panels evaluating such applications.^103^ Funders convene panels from different disciplines and with cross-disciplinary experience.^95^ Funders may need to allow training for research management staff and review panels,^75^ and to encourage applicants to justify their cross-disciplinary approach in their applications.^104^

Funders’ access to a large network of researchers means that they are able to link researchers across disciplines.^36 89 101 102^ Promotion of cross-disciplinary research can be done through engaging with universities and publishers for better recognition of, and opportunities for, cross-disciplinary research,^95^ promoting key success stories of cross-disciplinary research^95^ and engaging policy-makers when the research is policy relevant.^75^

## Discussion

This narrative literature review synthesised practical actions for fostering cross-disciplinary research, aiming to inform the design and implementation of cross-disciplinary global health research. Our review focused on empirical studies (either original research or research notes articles) that described practical actions to foster cross-disciplinary research whereas previous reviews^20 33 46–48^ tended to focus predominantly on theoretical articles.

The original research papers and research notes articles included in our review were almost all published in the last 10 years indicating a recent, and increasing, interest in understanding how to make cross-disciplinary research more effective. The majority of publications and their authors were from high-income countries, which is consistent with the findings of other reviews.^20 48^ A high-income country evidence base may reflect current cross-disciplinary research funding priorities.^20^ However, such a focus makes it difficult to generalise the findings to low-and-middle-income countries where there is a great need for cross-disciplinary research to tackle complex challenges.^105 106^ Applicability to the global health context where research is often conducted in North–South collaborations is also difficult.^107–109^ Qualitative research approaches were the predominant methods used, probably because such approaches are more appropriate than quantitative methods for gaining an in-depth and interpreted understanding of cross-disciplinary research,^110^ though such methods may be under-appreciated.^20 111^

### Preparing for the uncertainty and risk in cross-disciplinary research

This review identified personal attributes such as being receptive to new ideas, tolerating ambiguities as individual-level enablers for cross-disciplinary research. Those attributes apply to cross-disciplinary research teams, academic institutions and funders. Cross-disciplinary research is innovative and risky for all involved^37 60 61^ due to the uncertainty in research processes and outcomes.^21 32 33^ Such uncertainty mainly concerns the social and cognitive integration at individual, research theme and team/programmatic levels.^75^

### Leadership, team and community building for cross-disciplinary research

The team/programmatic actions for fostering cross-disciplinary research identified through this review are closely linked to leadership, management process, collaboration and teamwork,^24 25 28 33 112–127^ and reflect the proposed greater use of management science in global health.^44^

Our findings highlight the importance of mentoring and empowering early-career researchers who act as brokers and provide momentum for cross-disciplinary research, especially in generating new enquiries, collaborations and publications. Engaging in cross-disciplinary research requires competencies and actions in expanding knowledge and skills in multiple disciplines, sharing knowledge, listening, discussing, clarifying and building trust. Mentoring facilitates the nurturing of these competences.^128^ Previous contacts and working relationships were frequently mentioned as enablers for cross-disciplinary research collaborations and can be facilitated by experienced researchers and leaders.

**Figure 3:**
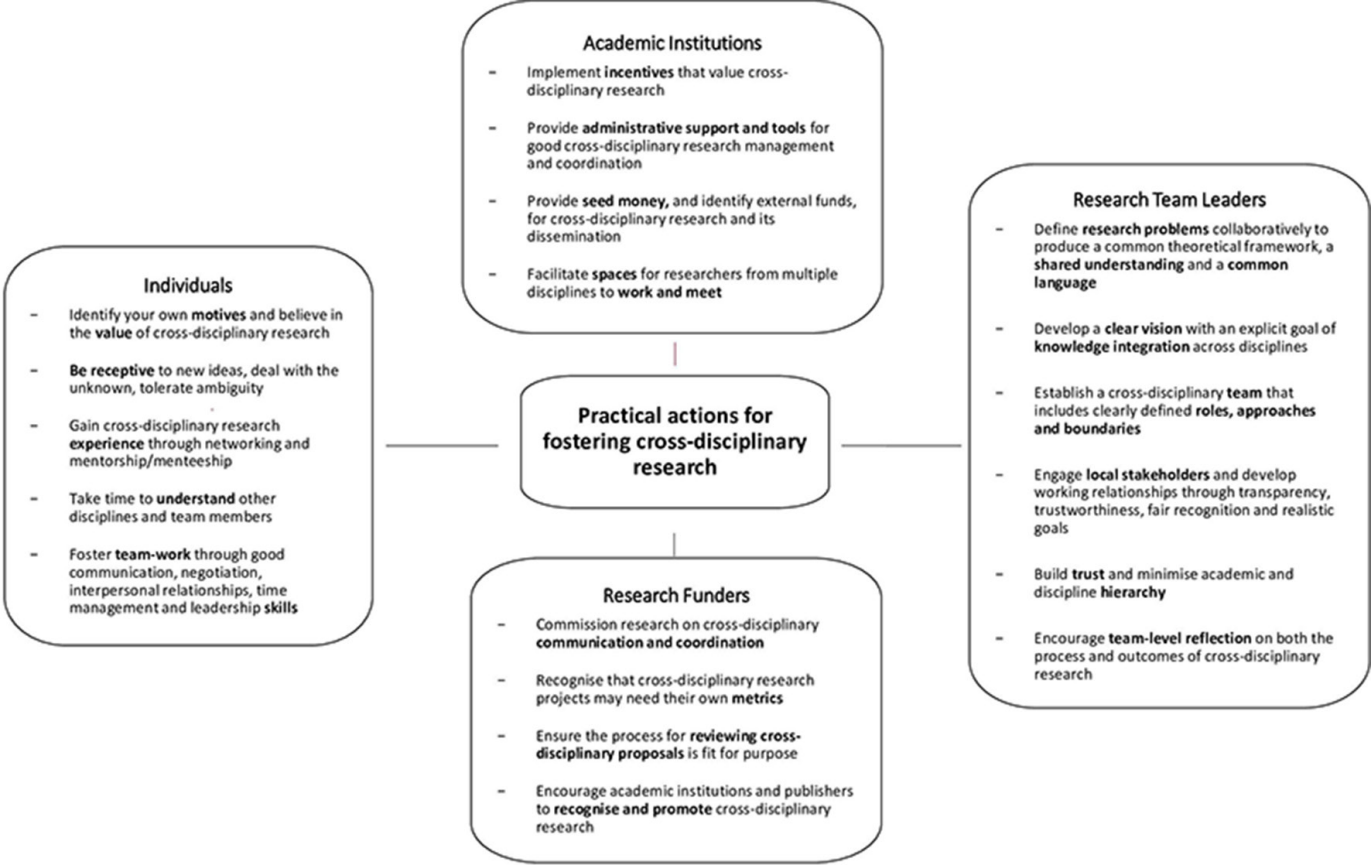
Practical actions for fostering cross-disciplinary research.

Cross-disciplinary research teams and their leaders should act as a driving force for the institutional changes in rewarding cross-disciplinary research teams and members.^129 130^ To attract, develop and maintain those who are or who have the potential to be good cross-disciplinary researchers, rewards for individuals engaging in cross-disciplinary research need to be enhanced.^40^ Figure 3 summarises practical actions for fostering cross-disciplinary research.

### Interactions across different levels

The practical actions at different levels identified do not exist in isolation, but rather influence each other. When funders provide long-term funding with a flexible management style, it is possible for cross-disciplinary research teams/programmes to spend time defining shared research problems,^61 68 75 95^ and to have time to develop working relationships and trust.^91 93^ When institutions reflect cross-disciplinary research teamwork appropriately in their incentives, it motivates cross-disciplinary research teams to take knowledge integration as an explicit goal^59^ and to appreciate research synergies.^38 54^ It also increases individual commitment and confidence in cross-disciplinary research teamwork.^40^ When a cross-disciplinary research programme has built a supportive cross-disciplinary research community, individuals can better deal with the unknown.^38 65^ When individuals are receptive to new ideas and learn from each other, there is a better chance for such teams to develop shared understandings trust.^38 51 55 60 62^

### Limitations

It is possible that not all relevant publications were included if they were unpublished or not indexed in the five databases we searched. To overcome this limitation, we scanned the reference lists of included publications for relevant articles. We used the term cross-disciplinary research to encompass multi-disciplinary, inter-disciplinary and trans-disciplinary research and frame our work to cover these three typologies. It is not possible to differentiate them in the literature consistently due to lack of agreement on their definitions. Still, we acknowledge that multi-disciplinary, inter-disciplinary and trans-disciplinary research are different. The quality of the included studies was not assessed, and there is a paucity of publications that explore enablers at socio-political level.

### Recommendations on future research

Our study findings indicate that critical knowledge gaps on how to foster cross-disciplinary research exist for low-and-middle-income countries and at a socio-political level. These gaps relate to leadership, management and teamwork: for example, how to develop an integrative and clear vision within cross-disciplinary teams, how to arrive at a common conceptual framework for cross-disciplinary research, how to practice team-level reflection on the process and outcomes of cross-disciplinary research, how to design and implement academic tenure and promotion schemes that reward teamwork instead of individual research, and how funders could enable innovation and flexibility within projects on cross-disciplinary research while ensuring accountability. Exploring the association between the actions that foster cross-disciplinary research and the quality of such research would be important for improving future global health research programmes.

## Conclusion

Our review found substantial evidence, particularly from high-income countries, that a wide range of practices could improve cross-disciplinary research in global health. There is very little evidence about whether these practices are appropriate and workable for low-and-middle-income countries. Critical knowledge gaps still exist around how leadership, management and teamwork processes can better integrate expertise from different disciplines to make cross-disciplinary research more effective.
