# Supplementary material for: Practical actions for fostering cross-disciplinary global health research: lessons from a narrative literature review
Source: BMJ Glob Health. 2020 Apr 30;5(4):e002293. doi: 10.1136/bmjgh-2020-002293 (PMC7213812; doi:10.1136/bmjgh-2020-002293)
Supplement: Supplementary data [file bmjgh-2020-002293supp001.pdf]

Supplementary File 1

The countries of authors and co-authors affiliations	N
**Developed countries**[1]<br><br>NB: Some studies had authors based in more than one country	63
USA	29
UK	16
Germany	8
Australia	4
New Zealand	3
Canada	2
Austria	2
Switzerland	2
The Netherlands	2
Belgium	1
Finland	1
Norway	1
Slovenia	1
Sweden	1
The countries where the studies took place	
**Developed countries**<br><br>NB: Some research projects were implemented in more than one country	51
USA	23
UK	11
Australia	3

[1] https://www.un.org/en/development/desa/policy/wesp/wesp_current/2014wesp_country_classification.pdf

Ding Y, *et al. BMJ Global Health* 2020; 5:e002293. doi: 10.1136/bmjgh-2020-002293

Canada	2
Germany	2
New Zealand	2
The Netherlands	2
Austria	1
Chile	1
Finland	1
France	1
Greece	1
Slovenia	1
Sweden	1
Switzerland	1
**Developing countries**<br><br>NB: Some research projects were implemented in more than one country	3
Brazil	3
Bangladesh	1
Bolivia	1
Burkina Faso	1
Ecuador	1
India	1
**Not specified**	4
Data collection methods (multiple methods may be adopted per study)	
Interviews	32
Survey	19
Observation	13

Focus group discussions	6
Workshop/group brainstorming	6
Desk research (combined with any of the above methods)	16
Others (i.e. participatory research evaluation by research members)	4
Not specified	22
Data analysis methods (multiple methods may be adopted per study)	
Thematic analysis	17
Descriptive analysis	11
Constant comparative method	5
Content analysis (2 were semi-quantified and others not specified)	6
Other quantitative analysis methods (including a confirmatory factor analysis, ordinary least squares regression, a mediation analysis, ordinal regression models and non-parametric Kruskal-Wallis test)	
Other qualitative analysis methods (including strategic mapping using Banxia Decision Explorer, a discourse analysis approach, and a grounded theory approach, developmental analytical approach)	4
bibliometric analysis	1
Not specified	29
Cross-disciplinary research term (multiple terms may be used per study)	
Multi-disciplinary research	11
Inter-disciplinary research	30
Trans-disciplinary research	26
Cross-disciplinary research	9
Provided definitions of multi-/inter-/trans-/cross-disciplinary research	42

Ding Y, *et al. BMJ Global Health* 2020; 5:e002293. doi: 10.1136/bmjgh-2020-002293
